# The algebraic extended atom-type graph-based model for precise ligand–receptor binding affinity prediction

**DOI:** 10.1186/s13321-025-00955-z

**Published:** 2025-01-22

**Authors:** Farjana Tasnim Mukta, Md Masud Rana, Avery Meyer, Sally Ellingson, Duc D. Nguyen

**Affiliations:** 1 Department of Mathematics, University of Kentucky, Lexington, KY 40506 USA; 2 Department of Mathematics, Kennesaw State University, Kennesaw, GA 30144 USA; 3 Division of Biomedical Informatics, College of Medicine, University of Kentucky, Lexington, KY 40506 USA; 4 Department of Mathematics, University of Tennessee, Knoxville, TN 37996 USA

**Keywords:** Algebraic graph learning, Extended atom type, Similarity computation, Non-redundant training sets, Protein-ligand interactions, Binding affinity predictions

## Abstract

Accurate prediction of ligand-receptor binding affinity is crucial in structure-based drug design, significantly impacting the development of effective drugs. Recent advances in machine learning (ML)–based scoring functions have improved these predictions, yet challenges remain in modeling complex molecular interactions. This study introduces the AGL-EAT-Score, a scoring function that integrates extended atom-type multiscale weighted colored subgraphs with algebraic graph theory. This approach leverages the eigenvalues and eigenvectors of graph Laplacian and adjacency matrices to capture high-level details of specific atom pairwise interactions. Evaluated against benchmark datasets such as CASF-2016, CASF-2013, and the Cathepsin S dataset, the AGL-EAT-Score demonstrates notable accuracy, outperforming existing traditional and ML-based methods. The model’s strength lies in its comprehensive similarity analysis, examining protein sequence, ligand structure, and binding site similarities, thus ensuring minimal bias and over-representation in the training sets. The use of extended atom types in graph coloring enhances the model’s capability to capture the intricacies of protein-ligand interactions. The AGL-EAT-Score marks a significant advancement in drug design, offering a tool that could potentially refine and accelerate the drug discovery process.

**Scientific Contribution**

The AGL-EAT-Score presents an algebraic graph-based framework that predicts ligand-receptor binding affinity by constructing multiscale weighted colored subgraphs from the 3D structure of protein-ligand complexes. It improves prediction accuracy by modeling interactions between extended atom types, addressing challenges like dataset bias and over-representation. Benchmark evaluations demonstrate that AGL-EAT-Score outperforms existing methods, offering a robust and systematic tool for structure-based drug design.

**Supplementary Information:**

The online version contains supplementary material available at 10.1186/s13321-025-00955-z.

## Introduction

In the rapidly evolving field of structure-based drug design, the precise prediction of ligand-receptor binding affinity stands as a cornerstone of success [1–4]. This critical aspect determines the efficacy of a drug (ligand) in interacting with its target, typically a protein, shaping the outcome of drug discovery processes. Central to advancing these predictions is the application of graph theory, a vital branch of discrete mathematics that provides a structured framework for modeling complex relationships in molecular interactions [5–12].

Graph theory, with its diverse branches including geometric, algebraic, and topological graph theory, has revolutionized the way scientists approach ligand-receptor binding affinity. Geometric graph theory focuses on spatial connectivity, capturing the essence of geometric relationships within molecular structures [13, 14]. Algebraic graph theory, on the other hand, delves into the realm of algebraic connectivity, utilizing matrix representations like adjacency and Laplacian matrices to elucidate molecular interactions [15, 16]. Topological graph theory bridges graphs and topological spaces, offering insights into the more abstract aspects of molecular configuration [17, 18]. These methodologies have not only enhanced drug discovery but also found widespread applications in biomedical sciences [19–21], chemical analysis [22–25], molecular property evaluation [26, 27], and drug repurposing [28, 29].

Scoring functions (SFs) are computational methods used to evaluate protein-ligand interactions and are crucial in structure-based drug design for differentiating between viable and non-viable hypotheses. These scoring functions, based on their theoretical underpinnings, can generally be categorized into the following types: physics-based scoring functions [30–34], empirical scoring functions [35–37], knowledge-based scoring functions [38, 39], and the increasingly prominent machine learning (ML)-based scoring functions [40, 41]. ML-based SFs, in particular, have garnered attention for their superior performance, driven by extensive datasets, comprehensive molecular descriptors, and advanced machine learning algorithms. However, the efficacy of these functions is often contingent on the size of the training set and the similarity between the training and test sets, a challenge that has been the focus of several recent studies [42–47].

Significant strides in developing machine learning-based scoring functions have been made by utilizing three distinct types of descriptors. These include physics-based descriptors, which cover aspects like electrostatic binding energies and atomic interactions (Coulombic and van der Waals) [48]; descriptors based on geometric graph theory [13]; and those derived from algebraic topology [49]. The core idea behind these methodologies is the assumption that the essential physical phenomena are typically found within low-dimensional spaces or manifolds, even though they exist in a broader, high-dimensional data space. This concept, while recognized in the field of manifold learning, presents a major challenge: effectively translating critical physical information from a high-dimensional context into a practical, low-dimensional format for molecules and their complexes. A notable approach to tackle this challenge is the application of multiscale weighted colored subgraphs (MWCS) [50]. In this approach, a protein’s structural graph is colored based on the types of interactions between its nodes, leading to the formation of distinct subgraphs. This method stands out for its simplicity, low-dimensional nature, and robustness. A key advantage is its minimal data input requirements for binding affinity predictions, which only need atomic names and coordinates. This simplicity allows the method to circumvent complex data processing and parameterization steps, eliminating the need for molecular mechanical force fields, like charges, bond measurements, van der Waals parameters, and others. This not only streamlines the process but also reduces errors often associated with parameterization.

In our prior research [14], we developed the ^sybylGGL^-Score, a sophisticated geometric graph-based method using extended multiscale weighted colored subgraphs for protein-ligand complexes. This approach leveraged graph coloring techniques based on protein atom names and ligand SYBYL atom types [51]. While ^sybylGGL^-Score demonstrated exceptional efficacy in predicting protein-ligand binding affinity, surpassing other advanced methods, it did not fully explore the potential of algebraic graph theory within the extended MWCS framework.

In our current study, we take this concept further by developing an algebraic graph-based MWCS with extended atom-type graph coloring, known as the AGL-EAT-Score. This model employs both the Laplacian and adjacency matrices to represent subgraphs, characterizing molecules and their interactions through eigenvalues and eigenvectors. The effectiveness of AGL-EAT-Score has been rigorously evaluated using benchmark datasets like CASF-2016, CASF-2013, and the Cathepsin S dataset. To enhance our model’s robustness, we conducted a similarity search to eliminate redundant complexes from our training sets, ensuring a more reliable analysis. A summary of the models used for performance comparison, including key features and algorithms, is presented in Table 1.

**Table 1:** Some state-of-the-art machine learning-based scoring functions

Method	Feature	Machine-learning algorithm
PIGNet [52]	Pairwise interactions via physics-informed equations	Neural networks
PerSpect-ML [18]	Algebraic topology/persistent spectral graph	GBT
ECIF::LD-GBT [53]	Protein-ligand atom-type pair counts considering each atoms connectivity and RDKit ligand descriptors	GBT
AGL-Score [15]	Algebraic graph	GBT
EIC-Score [54]	Differential geometry	GBT
RI-Score [50]	Geometric graph	Random forest
PLEC-nn [55]	Extended connectivity fingerprint	Neural networks
$\hbox {K}_{\textrm{DEEP}}$ [56]	3D voxel representation	CNN
Pafnucy [57]	3D voxel representation	CNN
TopBP [58]	Algebraic topology	GBT/CNN
$\Delta _{\textrm{Vina}} \hbox {RF}_{20}$ [59]	Autodock Vina and other physical features	Random forest
RF::VinaElem [60]	Intermolecular contacts and Autodock Vina features	Random forest
RF-Score [40]	Atom-type pair counts	Random forest

## Materials and methods

### Extended atom-type multiscale weighted colored subgraphs

In this section, we explore the development of comprehensive graph theory descriptors for a biomolecule or molecular complex. A biomolecular graph, denoted as ${\mathcal {G}}({\mathcal {V}}, {\mathcal {E}})$, is composed of vertices ${\mathcal {V}}$ and edges ${\mathcal {E}}$, providing a powerful tool for representing non-covalent interactions among atoms within the molecule. This graph theory representation is further enriched through the technique of graph coloring, which assigns distinct labels to various types of elements. This coloring process creates a graph that encodes different atomic interactions, enabling the construction of an inclusive and simplified representation of the dataset. Within this framework, atoms in the molecule, identified by these labels, are organized into subgraphs, and the colored edges signify element-specific interactions.

In our previous studies [13, 15], the classification of interactions relied on combinations of element symbols of protein-ligand atoms involved, such as C-O, C-N, etc. Following our most recent work [14], bipartite-colored subgraphs are defined for protein-ligand complexes, where graph coloring is based on extended atom types for proteins and SYBYL atom types for ligands. Protein atom types are identified by their names within the protein data bank (PDB) structure such as carbon alpha (CA), carbon beta (CB), carbon delta-1 (CD1), etc. These atom names serve as identifiers for specific positions within a protein’s three-dimensional arrangement. They help define the individual atoms that constitute amino acids, the building blocks of proteins, and provide crucial information about their spatial arrangement and chemical properties. A total of 37 distinct atom names are considered that are frequently found in protein structures within the PDB database. In ligand Tripos Mol2 structure, SYBYL atom types classify atoms based on their chemical attributes and surroundings within molecular structures, aiding in the identification of diverse atom categories, considering factors like hybridization state, bonding characteristics, and potential interactions. The incorporation of SYBYL atom types enables precise classification, including distinct subtypes for Carbon (C) elements, such as C.1, C.2, C.3, C.ar, and C.cat. The set ${\mathcal {A}}_p$ represents atom names of proteins,

1 $$\begin{aligned} {\mathcal {A}}_p&= \{C, CA, \ldots  , N, ND1, \ldots  , O,OD1, \ldots  , SD, SG\} \end{aligned}$$

And, the set ${\mathcal {A}}_l$ represents atom types of ligands,

2 $$\begin{aligned} {\mathcal {A}}_l&= \{As, B, Be, \ldots  , C.1, C.2, \ldots  , N.1, N.2, \ldots  , V, Zn\} \end{aligned}$$

For convenience, we define ${\mathcal {A}}$ as the collection of all atom types within a given molecular dataset as described above, where ${\mathcal {A}}_k$ denotes the atom type at the *i*th position within the set. We further symbolize the subgraph vertices as ${\mathcal {V}}$, which are characterized by the atom coordinates $r_i$ and their corresponding atom types $\alpha _i$:

3 $$\begin{aligned} {\mathcal {V}}=\{ ({\textbf{r}}_i,\alpha _i)| {\textbf{r}}_i\in {\mathbb {R}}^3; \alpha _i\in {\mathcal {A}}; i=1,2,\cdots ,N\} \end{aligned}$$

Additionally, we symbolize the subgraph edges as ${\mathcal {E}}$ and defined as follows:

4 $$\begin{aligned} {\mathcal {E}}&= \{ \Phi (\Vert {{\textbf{r}}_i-{\textbf{r}}_j}\Vert ;\eta _{kk'})| \alpha _i={\mathcal {A}}_k,\, \alpha _j = {\mathcal {A}}_{k'}; \nonumber \\&\quad i,j=1,2,\cdots ,N;\, \Vert {{\textbf{r}}_i-{\textbf{r}}_j}\Vert \le c \}, \end{aligned}$$

where $\Vert {{\textbf{r}}_i-{\textbf{r}}_j}\Vert$ denotes the Euclidean distance between the *i*th and *j*th atoms, and *c* represents a specified cutoff distance that defines the binding site between the atoms of types ${\mathcal {A}}_k$ and ${\mathcal {A}}_{k'}$. While *c* is a learnable parameter that can be optimized through cross-validation, we have chosen $c=12$ Å  for this work, as it has proven effective in our previous studies [14, 61] and is also utilized in RF-Score [62]. We calculate the edge weights based on the characteristics distance $\eta _{kk'}$ between pairs of atom types ${\mathcal {A}}_k$ and ${\mathcal {A}}_{k'}$ using the subgraph weight function $\Phi$. The weight function $\Phi$ assesses the interaction strength between atoms, taking into account their Euclidean distances, and it satisfies the following conditions:

5 $$\begin{aligned} \Phi (\Vert {{\textbf{r}}_i-{\textbf{r}}_j}\Vert ;\eta _{kk'})&=1, \quad \textrm{as}\; \Vert {{\textbf{r}}_i-{\textbf{r}}_j}\Vert \rightarrow 0,\nonumber \\ \Phi (\Vert {{\textbf{r}}_i-{\textbf{r}}_j}\Vert ;\eta _{kk'})&=0, \quad \textrm{as}\; \Vert {{\textbf{r}}_i-{\textbf{r}}_j}\Vert \rightarrow \infty , \alpha _i={\mathcal {A}}_k,\, \alpha _j = {\mathcal {A}}_{k'}. \end{aligned}$$

Often, a popular selection for $\Phi$ is the generalized exponential function or the generalized Lorentz function denoted as follows:

6 $$\begin{aligned} \Phi _E(\Vert {{\textbf{r}}_i-{\textbf{r}}_j}\Vert ;\eta _{kk'}) = e^{-(\Vert {{\textbf{r}}_i-{\textbf{r}}_j}\Vert /\eta _{kk'})^\kappa }, \quad \kappa >0, \end{aligned}$$

and

7 $$\begin{aligned} \Phi _L(\Vert {{\textbf{r}}_i-{\textbf{r}}_j}\Vert ;\eta _{kk'}) = \frac{1}{1+\left( \Vert {{\textbf{r}}_i-{\textbf{r}}_j}\Vert /\eta _{kk'}\right) ^\kappa }, \quad \kappa >0, \end{aligned}$$

The generated weighted colored subgraph ${\mathcal {G}}({\mathcal {V}}, {\mathcal {E}})$ offers a robust depiction of molecular attributes at the atomic scale. Analyzing this subgraph allows us to extract detailed molecular descriptors and explore the system’s multiscale behavior. This behavior is a result of considering various characteristic distances $\eta _{kk'}$ for different atom type pairs, enabling the creation of diverse and scalable graph-based descriptors, including the geometric subgraph centrality (GSC), defined as

8 $$\begin{aligned} \textrm{GSC}(\eta _{kk'})&=\sum _i \mu _i(\eta _{kk'})=\sum _i \sum _j \Phi (\Vert {{\textbf{r}}_i-{\textbf{r}}_j}\Vert ;\eta _{kk'}),\nonumber \\&\quad \alpha _i = {\mathcal {A}}_{k},\, \alpha _j = {\mathcal {A}}_{k'}, \end{aligned}$$

This extends the concept of the bipartite subgraph we utilized in our prior research on predicting protein-ligand binding affinities and free energy ranking [13], where every edge connects an atom in the protein with an atom in the ligand. The matrix representations of such subgraphs offer a simple and expressive way to describe the interactions between subgraph elements using matrices. As following our previous study [15], we utilized two highly significant matrices: the Laplacian matrix and the adjacency matrix in our present work.

For each atom-type pair subgraph, we compute the Laplacian matrix $L\left( \eta _{k k^{\prime }}\right)$, which is defined as follows:

9 $$\begin{aligned} L_{i j}\left( \eta _{k k^{\prime }}\right) =&{\left\{ \begin{array}{ll}-\Phi \left( \left\| {\textbf{r}}_i-{\textbf{r}}_j\right\| ; \eta _{k k^{\prime }}\right) &  \text{ if } i \ne j, \alpha _i={\mathcal {A}}_k, \alpha _j={\mathcal {A}}_{k^{\prime }} \\ &  \text{ and } \left\| {\textbf{r}}_i-{\textbf{r}}_j\right\| \le c; \\ -\sum _j L_{i j} &  \text { if } i=j\end{array}\right. } \end{aligned}$$

It’s important to emphasize that all eigenvalues of the Laplacian matrix are nonnegative. Let us define the eigenvalues and eigenvectors of $L\left( \eta _{k k^{\prime }}\right)$ as ${\lambda _j}^L$,$j = 1,2,\ldots$ and ${u_j}^L$,$j = 1,2,\ldots$.

For each atom-type pair subgraph, we also compute the adjacency matrix $A\left( \eta _{k k^{\prime }}\right)$, which is defined as follows:

10 $$\begin{aligned} A_{i j}\left( \eta _{k k^{\prime }}\right) =&{\left\{ \begin{array}{ll}-\Phi \left( \left\| {\textbf{r}}_i-{\textbf{r}}_j\right\| ; \eta _{k k^{\prime }}\right) &  \text{ if } i \ne j, \alpha _i={\mathcal {A}}_k, \alpha _j={\mathcal {A}}_{k^{\prime }} \\ &  \text{ and } \left\| {\textbf{r}}_i-{\textbf{r}}_j\right\| \le c; \\ 0 &  \text { if } i=j\end{array}\right. } \end{aligned}$$

Indeed, eigenvalue analysis is widely recognized as a computationally expensive task. However, our AGL-EAT-Score approach benefits from two crucial factors that enhance its computational efficiency. Firstly, we restrict matrix constructions to encompass solely those atoms located in the proximity of the protein-ligand binding site. To determine the binding site of the protein-ligand complex, we define a cubic domain extending 12 Å  from the ligand atoms. This approach involves selecting protein atoms that fall within this defined cubic region by applying the cutoff distance to the ligand’s maximum and minimum coordinates along each axis. Furthermore, our atom-type-specific criteria further narrow down the atoms involved in each matrix construction. Consequently, we work with numerous small matrices, which enables an efficient spectral approach for analyzing protein-ligand binding affinities.

### Algebraic graph learning

We employed Machine learning algorithms to analyze the eigenvalue statistics descriptors generated from the weighted colored subgraph Laplacian matrix or adjacency matrix as discussed above. For a given protein-ligand complex, we considered 37 unique atom names in the protein and 45 SYBYL atom types for the ligand for graph coloring, resulting in $37\times 45=1665$ unique colored subgraphs. From each subgraph, we extracted nine statistics of the positive eigenvalues: the sum, mean, median, minimum, maximum, standard deviation, variance, number of positive eigenvalues, and the sum of the squares of the positive eigenvalues, as well as the edge counts of the subgraph. This yielded a total of $1665\times 10=16650$ features for a complex. These extracted features will map the high-dimensional structures of biomolecular complexes into low-dimensional representations while preserving the vital physical and chemical properties of the complexes.

Supervised machine learning algorithms can include both classification and regression tasks, the labeled dataset is divided into two subsets: a training set and a test set. Let us denote ${\mathcal {G}}({\mathcal {X}}_i, \lambda )$, a function encoding the geometric information of a molecule into suitable graph representations using ${\mathcal {X}}_i$, a labeled dataset corresponding to the *i*th data point in the training set and $\lambda$, a set of kernel parameters. The following loss minimization problem further reformulates the optimization process for training a machine learning model,

11 $$\begin{aligned} \min _{\lambda , \theta } \sum _{i\in I} {\mathcal {L}}({\textbf{y}}_i,{\mathcal {G}}({\mathcal {X}}_i, \lambda ); \theta ) \end{aligned}$$

**Fig. 1:**
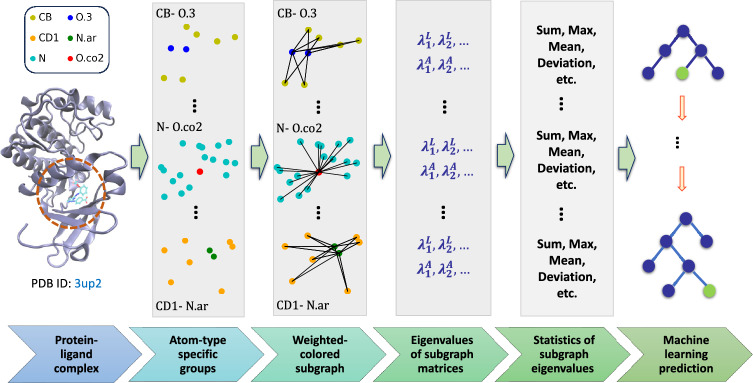
Visualization of the AGL-EAT graph learning approach. First column: binding site of the molecular complex identified by its PDBID: 3up2. Second column: three different kinds of protein-ligand atom-type pairs. Third column: weighted colored subgraph representations of the corresponding atom-pairs. Fourth column: eigenvalues of the subgraph Laplacian and adjacency matrices. Fifth column: various statistics of these eigenvalues. Fifth column: advanced machine learning models like gradient boosting trees to combine and process these statistics for training and making predictions

Here, ${\mathcal {L}}$ denotes a scalar loss function that needs to be minimized, and ${\textbf{y}}_i$ refers to the labels assigned to the *i*th sample in the training set *I*. The set $\theta$ includes hyperparameters that are dependent on the chosen machine learning algorithm and are usually tuned to achieve optimal performance. While various machine learning algorithms, including random forest, gradient boosting trees, graph neural networks, and convolutional neural networks, can be applied alongside the graph descriptors introduced in our study, our primary focus is to assess the effectiveness of the proposed algebraic graph features. To achieve this, we emphasize the use of gradient boosting trees (GBTs) as a regression model, a machine learning algorithm recognized for its robustness against overfitting. The 16,650 features extracted from the eigenvalues of multiscale weighted colored subgraphs of a protein-ligand complex serve as the input to the regression model, while the output corresponds to the predicted binding affinity. The visual depiction of our algebraic graph-based learning approach is presented in Fig. 1.

In our study, the implemented GBDT module in scikit-learn version 0.24.1 with the parameters, $\texttt {n\_estimators}=20 000$, $\texttt {max\_depth} = 8$, min_samples_split = 2, learning_rate = 0.005, loss = ls, subsample = 0.7, and max_features = sqrt.

### Datasets

To assess the validity of our proposed model, we employed two widely acknowledged PDBbind benchmark datasets: CASF-2013 and CASF-2016, and the CatS dataset, which is part of the D3R datasets, a global grand challenge in drug design.

The PDBbind database offers an extensive repository of experimentally determined binding affinity data for biomolecular complexes found within the Protein Data Bank (PDB) [63]. Every PDBbind benchmark dataset comprises three intersecting subsets: the general set, the refined set, and the core set, with the core set being a subset of the general set and the refined set. The core set serves as the testing dataset for the respective benchmark. More details of the PDBbind datasets can be explored in the PDBbind website http://www.pdbbind.org.cn/.

On the other hand, the Drug Design Data Resource (D3R) Grand Challenge [64, 65] focuses on specific datasets, each of which includes a single protein and multiple ligands, all accompanied by measured affinity data. The Cathepsin S (CatS) dataset of D3R [65] consists of 459 CatS inhibitors for binding affinity prediction. Detailed information about this dataset can be found on the official D3R website https://drugdesigndata.org/about/grand-challenge-4/cathepsin_s. A summary of all the datasets used in this study has been listed in Tables 2 and 3.

**Table 2:** Summary of PDBbind datasets used to validate our model

Datasets	Training sets		Test set
	Refined set	General set	Core set
CASF-2013 benchmark	3516	11713	195
CASF-2016 Benchmark	3772	12998	285

**Table 3:** Summary of CatS dataset used to validate our model

Dataset	Size of training set	Size of test set
CatS	431	459

### Evaluation metrics

In this research, we evaluated the performance of our model’s scoring power through various metrics, including including root mean squared error (RMSE), mean absolute error (MAE), standard deviation (SD), as well as Pearson’s Correlation Coefficient, Kendall’s Tau, and Spearman’s Rho between the experimental and predicted pK values. Pearson’s Correlation Coefficient measures the linear relationship between two variables, ranging from -1 to 1, where 1 indicates a perfect positive linear relationship, -1 indicates a perfect negative linear relationship, and 0 indicates no linear relationship, thereby assessing how well the model’s predictions correlate with actual values. Kendall’s Tau measures the ordinal association between two variables by evaluating the strength and direction of the association through concordant and discordant pairs, making it useful for datasets with ordinal data or non-linear relationships. Spearman’s Rho assesses the monotonic relationship between two variables, similar to Pearson’s but based on rank order rather than actual values, making it suitable for evaluating models where the relationship may not be strictly linear but still monotonic.

For comparison purpose, we employed Pearson’s Correlation Coefficient for the PDBbind datasets, while for the CatS dataset, we utilized both Kendall’s Tau and Spearman’s Rho. These specific metrics for each dataset were chosen based on their popularity and acceptance within the research community.

## Results and discussion

In this section, we present the results of hyperparameter optimization and the performance of our proposed AGL-EAT-Score on various benchmark datasets. Furthermore, we conduct a rigorous similarity test analysis to validate the robustness of AGL-EAT-Score. In our study, we adopt the notation $\text {AGL-EAT}_{\beta ,\kappa ,\tau }^{{\mathcal {B}}}$ to characterize algebraic graph learning for extended atom types features. Here, ${\mathcal {B}}$ represents the type of matrix used, specifically, ${\mathcal {B}} =\textrm{Adj}$ denotes the adjacency matrix, while ${\mathcal {B}} = \textrm{Lap}$ refers to the Laplacian matrix. The parameter $\beta$ indicates the specific kernel types, while $\kappa$ and $\tau$ correspond to the respective kernel parameters. Specifically, we employ the generalized exponential kernel denoted by $\beta =\Phi _E$, and the generalized Lorentz kernel represented by $\beta = \Phi _L$, for generating AGL-EAT features. The parameter $\tau$ is a scaling factor determining the characteristic distance $\eta _{kk'}$ between atom type *k* and atom type $k'$. This distance is calculated as $\eta _{kk'}=\tau (r_k + r_{k'})$. Here, $r_k$ and $r_{k'}$ are the van der Waals radii of the atoms of type *k* and type $k'$, respectively.

### Hyperparameter optimization

Hyperparameter optimization of a machine learning model involves the search for the most suitable combination of hyperparameter values that yield the best performance on a specific dataset, all within a reasonable time frame. To make our AGL-EAT-Score model work best for each benchmark, we optimize two key parameters $\kappa$ and $\tau$ for a given kernel type $\beta$ and matrix type ${\mathcal {B}}$. We use a five-fold cross-validation (CV) along with a grid search method to find the best values for $\tau$, which we search within the range of 0.5 to 10, and for $\kappa$, within the range of 0.5 to 20. We increment both parameters by 0.5 in the search. Higher values for the power parameter $\kappa$ are chosen to approximate the ideal low-pass filter (ILF) [13].

We applied a five-fold cross-validation on the refined set excluding the core set to find the optimized kernel parameters for each of the PDBbind benchmark datasets. We train our model on both the PDBbind refined set and general set using the derived optimized hyperparameters and then evaluate the model’s performance on the corresponding test set. However, for the CatS dataset, we perform a five-fold cross-validation on the training set and evaluate the model’s performance on the CatS test set provided by the D3R database. A detailed discussion of optimized hyperparameters and the model’s performances on each of the datasets used in this study has been documented in Figs. S1, S2, and S3 in the Supporting Information.

### CASF-2016 benchmark

For the CASF-2016 benchmark, the optimal kernel parameters with the Adjacency matrix are $$\beta =\Phi _E$$, $$\kappa =16.5$$, and $$\tau =3.0 $$ with a median Pearson’s correlation coefficient $$R_p =0.796 $$ and the optimal kernel parameters with the Laplacian matrix are $$\beta =\Phi _E$$, $$\kappa =19.5$$, and $$\tau =2.5 $$ with a median $$R_p$$ of 0.795.

After the best models have been identified for each benchmark, our goal is to assess their performance on the test set by calculating Pearson’s correlation coefficient between the predicted and experimental binding affinities. We first train each model using the refined set, and then make predictions on the test set. To ensure reliable predictions, we repeat this process up to 50 times and compute the average of all predicted values to obtain the final predicted set. Next, we train the model using the general set, excluding the CASF-2016 core set. Training on this larger dataset, despite the lower quality 3D structures, will validate the robustness of the proposed models against more diverse and potentially irrelevant data. Similarly, we repeat the model training 50 times to generate predicted values, and these values are then averaged to produce the final prediction.

**Fig. 2:**
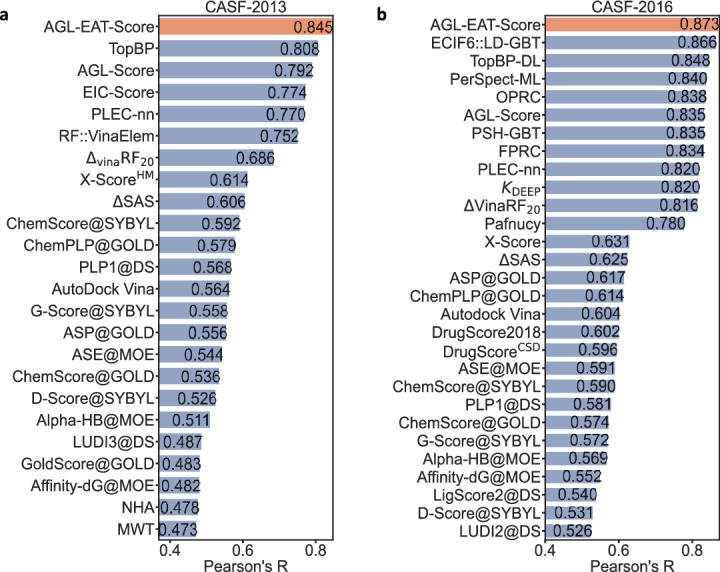
Performance comparison plot measured in Pearson’s correlation of our AGL-EAT-Score and other machine learning-based models on the **a** CASF-2013, and **b** CASF-2016 benchmark datasets. Our model AGL-EAT-Score (highlighted in red color) scores. In CASF-2013 benchmark, our model achieved $R_p = 0.845$ and the results of other methods obtained from prior research [59, 60, 66]. In CASF-2016 benchmark, our model achieved $R_p = 0.873$ and the results of other methods obtained from prior research [57, 59, 67]

A summary of the performances of the best AGL-EAT models on the CASF-2016 is presented in Table 4. The best model reported is $\text {AGL-EAT}_{\Phi _E,16.5,3.0}^{\textrm{Adj}}$ with an $R_p$ of 0.873. A comparison within the CASF-2016 benchmark is presented in Fig. 2b, showcasing our model’s superior performance as it ranks at the top among other models. The efficiency of the AGL-EAT-Score is also noteworthy, particularly regarding the running time for feature generation and model training. On average, generating features for a given protein-ligand complex takes less than one second. For model training, we utilized a high-performance computing cluster with one node, a single core, and a memory allocation of 10 GB, powered by an Intel(R) Xeon(R) Gold 6126 CPU running at 2.60 GHz. The total running time for training the AGL-EAT models with the PDBbind v2016 general set was approximately 50 min and 8 s, while training with the PDBbind v2016 refined set took about 12 min and 58 s. A comparison of models’ running time is provided in supplementary Table S1.

**Table 4:** Performance of various AGL-EAT-score models on CASF–2016 test set

Model	Trained with refined set				Trained with general set			
	RMSE	MAE	SD	R	RMSE	MAE	SD	R
$\text {AGL-EAT}_{\Phi _E,16.5,3.0}^{\textrm{Adj}}$	1.29	1.02	0.78	0.835	1.15	0.89	0.72	0.873
$\text {AGL-EAT}_{\Phi _E,19.5,2.5}^{\textrm{Lap}}$	1.28	1.02	0.78	0.837	1.15	0.90	0.72	0.871

Error metrics are in pKd unit

### CASF-2013 benchmark

Subsequently, we consider the CASF-2013 benchmark dataset from the PDBbind database. Since the CASF-2013 training set is smaller than the previously discussed CASF-2016 set, one might expect lower performance compared to the above results. However, the CASF-2013 benchmark will further confirm the robustness of the proposed model with less informative training data. The AGL-EAT model with the Adjacency matrix shows optimal kernel parameters $\kappa = 5.5$ and $\tau = 2.0$ with kernel type $\beta =\Phi _E$, resulting in a median Pearson’s correlation coefficient $R_p = 0.795$. On the other hand, the model with the Laplacian matrix has optimal kernel parameters $\kappa = 4.5$ and $\tau = 2.0$ with kernel type $\beta =\Phi _E$, delivering a median $R_p = 0.796$.

Once the top-performing models for this benchmark are identified, we evaluate the performance of our model on the corresponding test set by calculating Pearson’s correlation coefficient between predicted and experimental binding affinities. Following CASF-2016, a similar repetition of model training, totaling 50 cycles, both for the refined set and the general set is undertaken to generate predicted values, which are subsequently averaged to derive the final prediction. The performance summary of the top AGL-EAT models for the CASF-2013 benchmark is outlined in Table 5, with the $\text {AGL-EAT}_{\Phi _E,5.5,2.0}^{\textrm{Adj}}$ model achieving the highest Pearson correlation coefficient ($R_p = 0.845$). A visual comparison in the benchmark, depicted in Fig. 2a, demonstrates our model’s leading performance against competing models, underscoring its effectiveness in the evaluation.

**Table 5:** Performance of various AGL-EAT-Score models on CASF–2013 test set

Model	Trained with Refined Set				Trained with General Set			
	RMSE	MAE	SD	R	RMSE	MAE	SD	R
$\text {AGL-EAT}_{\Phi _E,5.5, 2.0}^{\textrm{Adj}}$	1.41	1.16	0.79	0.812	1.31	1.07	0.74	0.845
$\text {AGL-EAT}_{\Phi _E,4.5, 2.0}^{\textrm{Lap}}$	1.44	1.17	0.81	0.804	1.32	1.08	0.75	0.841

Error metrics are in pKd unit

### CatS dataset

In the context of the CatS dataset, we employ Kendall’s tau correlation coefficient as the performance evaluation metric, which assesses the model’s ability to capture the ranking and correlation of predicted binding affinities with the actual values, providing a comprehensive evaluation of the model’s performance. The optimal kernel parameters for the Adjacency matrix are $\kappa =12.5$ and $\tau = 8.0$ with exponential kernel type producing a median Kendall’s tau of 0.57837. The optimal kernel parameters with the Laplacian matrix are $\kappa =16.5$ and $\tau = 10.0$ for $\beta =\Phi _E$ with a median Kendall’s tau of 0.57305.

**Fig. 3:**
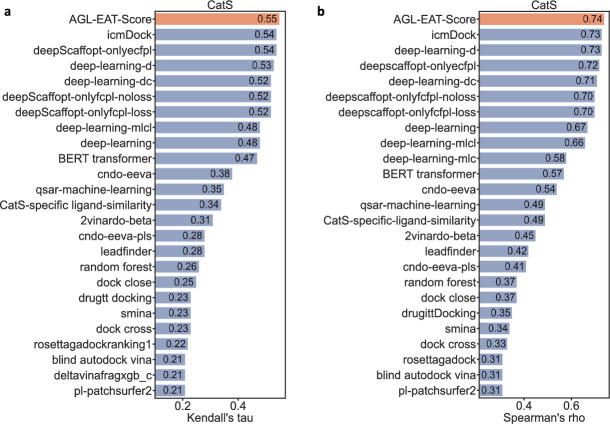
Performance comparison plot of our AGL-EAT-Score and other machine learning-based models on the Cathepsin S (CatS) dataset. Our model AGL-EAT-Score (highlighted in red color) scores Kendall’s $\tau = 0.55$ and Spearman’s $\rho = 0.74$

After having the best-optimized model for the CatS training set, we assess the performance on the test set by calculating Kendall’s tau correlation coefficient between the predicted and experimental binding affinities. We train each of these optimized models using the training set and subsequently generate predictions for the test set. We repeat this process up to 50 times and calculate the average of all predicted values to yield the final predicted set, from which we calculated Kentall’s tau correlation coefficient (Kendall’s $\tau$) and Spearman’s rho correlation coefficient (Spearman’s $\rho$). Table 6 reports the performance of our models for CatS dataset. The best-performing model for this dataset is the $\text {AGL-EAT}_{\Phi _E,5.5,2.0}^{\textrm{L}}$, achieving a Kendall’s tau of 0.552, and a Spearman’s $\rho$ of 0.742. Figure 3 provides a performance comparison between our AGL-EAT-Score and other machine learning-based models taken from the official results of the D3R GC4 [65]. The results show that our model outperforms the affinity ranking of 459 CatS compounds in terms of Kendall’s $\tau$ and Spearman’s $\rho$.

**Table 6:** Performance of various AGL-EAT-Score models on CatS data set

Model	RMSE	MAE	SD	Kendall’s $\tau$	Spearman’s $\rho$
$\text {AGL-EAT}_{\Phi _E,5.5, 2.0}^{\textrm{Adj}}$	0.41	0.31	0.26	0.539	0.729
$\text {AGL-EAT}_{\Phi _E,4.5, 2.0}^{\textrm{Lap}}$	0.40	0.31	0.25	0.552	0.742

Error metrics are in pKd unit

### Validation of the robustness of AGL-EAT model

#### Non-redundant training sets

The performance of machine learning scoring functions is known to be influenced by the size of the training set and the degree of similarity between the training set and the test set [68, 69]. However, robust and consistent machine learning-based scoring functions are expected to show a linear improvement with respect to the increment of relevant information in the training data [70–72]. To this end, we investigated our model’s performance on the multiple non-redundant training sets. These non-redundant training sets are datasets that have been carefully curated to ensure that the included complexes are distinct from the test set complexes and do not contain redundant or highly similar complexes with test complexes. These sets are designed to reduce bias and overfitting in machine learning models by providing diverse and representative training data.

#### Similarity computation

In our endeavor to construct a non-redundant training set, we explored the evaluation of three distinct similarity measurements between protein-ligand complexes. The first of these measurements, known as protein sequence similarity and denoted as $P_s$, quantifies the likeness between protein sequences. The second metric, $L_s$, deals with the structural resemblance of ligands. Lastly, the third measurement, $BS_s$, concerns itself with the comparison of protein-ligand binding sites. These similarity metrics collectively underpin the process of generating our non-redundant training set, allowing us to make informed decisions regarding redundancy reduction in the dataset. To compute the sequence similarity of two protein structures, we used $``ggsearch36''$ from FASTA (version 36.3.8) [73], which employs a global-global (Needleman-Wunsch) search algorithm. The ligand structure similarity was computed using ROCS (version 3.5.1.1) [74, 75], which employs a Gaussian function with smoothness characteristics to model the molecular volume, allowing for systematic optimization to achieve the most accurate global fit. The binding site similarity was computed using PocketMatch (version 2.1) [76, 77], which assesses the similarity of binding sites using structural descriptors like residue properties and interatomic distances. This tool can also provide atomic-level alignments derived from pairings of amino acid residues.

To initiate the search for non-redundant complexes, we eliminated the overlaps between the train and test sets. We will be adopting the terms “hard overlap” to refer to the complexes that overlap between the train set and test sets, and “soft overlap” to refer to the structurally similar complexes in the train set and test sets, as defined by Minyi Su et al [45].

**Fig. 4:**
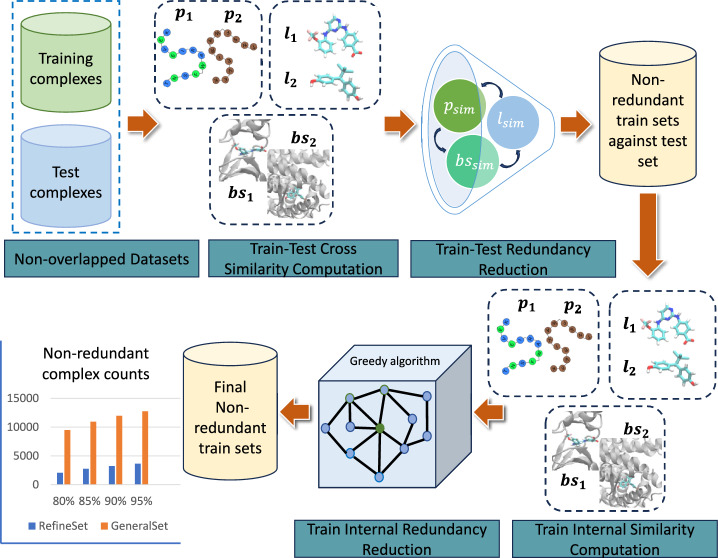
Visualization of the strategy for searching a non-redundant training set for different similarity cutoffs. In the initial phase, we conducted calculations to determine the cross-similarity between the training and test sets, followed by a process to reduce redundancy. In the following phase, we performed internal similarity calculations on the training set and subsequently reduced redundancy to get the final non-redundant training sets

The process of searching for non-redundant training sets has been illustrated in the following contexts and visually represented in Fig. 4.

1. **Training Set vs Test Set Similarity Evaluation:** We start by eliminating any “hard overlap” complexes shared between the training set and the test set. We then proceed to evaluate the similarity between every complex in the training set against every complex in the test set by employing the three similarity metrics, $P_s$, $L_s$ & $BS_s$ as discussed above.
2. **Training Set vs Test Set Redundancy Reduction:** A training complex was classified as redundant to the test set if all three similarity metrics were above the defined cutoff and further eliminated from the training set.
3. **Training Set Internal Similarity Evaluation:** Following that, we compute the similarities among the remaining complexes within the training set. If the measurements for all three similarity metrics between two complexes exceed the defined cutoff, we categorize these complexes as redundant to each other.
4. **Training Set Internal Redundancy Reduction:** Finally, we adopt a systematic approach to eliminate redundant samples from the training set to get the optimal training sets for different similarity cutoffs.

***PDBbind v2016:***

 In our study, we explored the calculation of similarity for both the PDBbind v2016 general set and the refined set. We derived non-redundant training sets for six distinct similarity cutoff points: $70\%$, $75\%$, $80\%$, $85\%$, $90\%$, and $95\%$. The summary of non-redundant complexes for different similarity cutoffs is listed in Table S2.

However, it’s important to note that a similar comparison for the PDBbind v2015 dataset wasn’t conducted since nearly all its molecules are already included in the PDBbind v2016 dataset, with only a marginal difference of around $10\%$.

***CatS:***

The CatS dataset has a limited size of training data, where the performance of the model largely depends on the quality of the information in the training data rather than its size. Therefore, we decided to explore the redundant complexes instead of non-redundant complexes, i.e. where multiple complexes shared common features or functions. Our approach to searching for redundant complexes in the CatS dataset closely paralleled the methodology we employed when investigating non-redundant complexes in the PDBbind dataset. We derived redundant training sets for 10 distinct similarity cutoffs, starting from 45 to 90%. The summary of redundant complexes for different similarity cutoffs for the CatS dataset is presented in Table S3.

Next, we evaluate the performance of our proposed model trained on these redundant datasets. By doing so, we aim to understand how well the model generalizes and performs across different levels of data redundancy. Redundant complexes often share similar features; however, the degree of similarity largely depends on the intrinsic information of the training data and its relevance to the test data. Therefore, we strive to uncover how the level of similarity between the training and test data affects the model’s accuracy.

#### Performances on PDBbind v2016 Non-redundant training sets

Indeed, it is widely recognized that the size of the training set and the degree of similarity between the training set and the test set have a profound impact on machine learning scoring functions [68, 69]. To this end, we aim to investigate our model’s performance on the multiple non-redundant training sets of PDBbind v2016 General set and Refined set with different levels of redundancy.

**Fig. 5:**
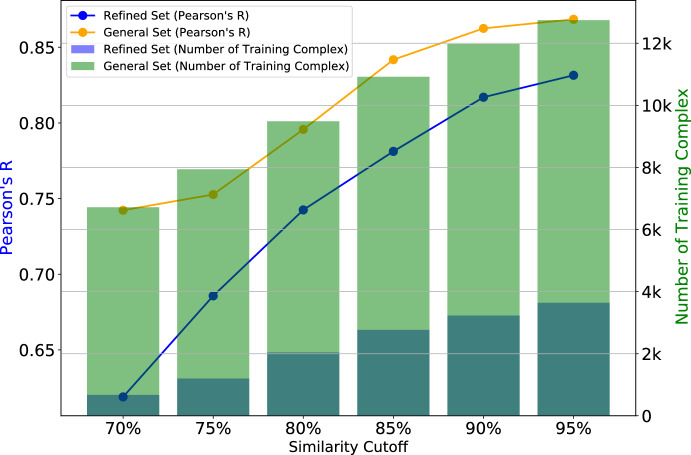
Performance comparison plot of non-redundant training sets of PDBbind v2016 RefinedSet and GeneralSet

Figure 5 visualizes the performance of our proposed model when trained on these non-redundant training sets. Interestingly, when we calibrate our model on a training set that shares a high level of similarity with the test set, for example, employing a $95\%$ similarity cutoff from the General set, we achieved a Pearson correlation of 0.869. This is remarkably close to the Pearson correlation of 0.873 obtained when using the complete training set. These findings emphasize the consistent and robust nature of our model across varying non-redundant training sets, without experiencing significant drops in predictive capabilities. This robust performance underscores the model’s reliability and versatility in handling diverse datasets.

Investigating the performances across various non-redundant training sets reveals another interesting fact: data quality significantly influences the model’s effectiveness. Consider the refined non-redundant training set for the $95\%$ similarity cutoff, notably smaller in size compared to the general non-redundant training set at the $70\%$ similarity cutoff. However, the performance of the smaller refined non-redundant set substantially outperforms the larger set. This observation underscores the importance of data quality over quantity, highlighting how the focused, refined data yield more accurate models despite their smaller scale.

#### Performances on CatS redundant training sets

**Fig. 6:**
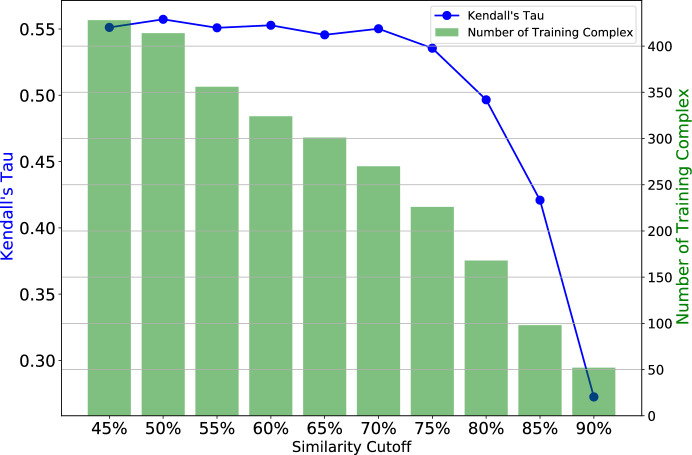
Performance comparison plot of redundant training sets of CatS Dataset

In exploring the CatS dataset’s redundant training complexes, we observed a nuanced impact of redundancy levels on our model’s performance. Training the model on sets with varying similarity cutoffs, we noted that higher redundancy maintained stable performance, as measured by Kendall’s Tau. As captured in Fig. 6, for $45\%$ similarity cutoff we achieved a Kendall’s Tau = 0.5512, which remains quite stable up to $75\%$ similarity cutoff with Kendall’s Tau = 0.5355. This stability suggests the model effectively leverages redundant training complexes without significant loss in predictive power. However, beyond this threshold, performance declined, highlighting the need to balance leveraging redundancy for rich feature extraction and avoiding diminishing returns from overly similar and less diverse training data. This emphasizes the importance of balancing diversity and similarity in collecting training samples.

#### Importance of extended atom types

We further performed an investigation by expanding our previous work, AGL-Score [15] to justify the impact of the protein-ligand extended atom type (EAT) features in the model’s performance. We follow a systematic approach throughout this investigation.

**Fig. 7:**
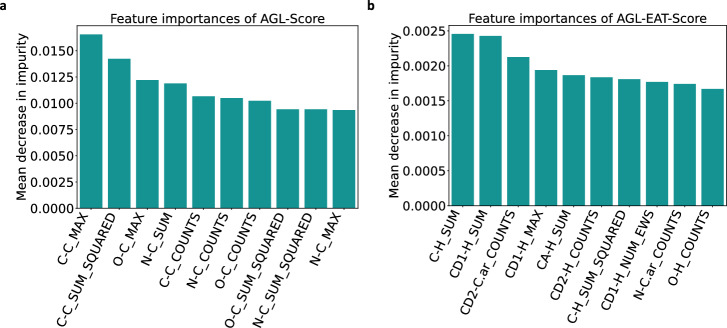
Feature importance of **a** AGL-Score [15] **b** our AGL-EAT-Score model on PDBbind v2016 general set using the mean decrease in impurity of the gradient boosting trees. The horizontal axis on the graph denotes the names of the features, which are represented as various statistical metrics (like sum, mean, median, and others) specific to the atom name groups in the ligand-protein interaction

We initiated the process by ranking the important features of both the AGL-EAT-Score and AGL-Score models, using the PDBbind v2016 general set features as a reference. Figure 7 illustrates the top ten important features of the AGL-EAT-Score and AGL-Score models in this dataset. As shown in Fig. 7a, some of the most important interactions of AGL-Score are C-C, O-C, N-C so on. For the first set of experiments, we replaced the C-C features in AGL-Score with the extended atom type features from AGL-EAT-Score, specifically the C-C.1, CA-C.1, CB-C.1, C-C.2, CA-C.2, CB-C.2, and so on. We then employed this modified feature set to predict the test dataset. To ensure the reliability of predictions, we repeated the process 50 times and obtained the final predicted set by averaging all the predicted values. In the case of the base AGL-Score model, we reported a performance of $R_p = 0.8559$ on the PDBbind v2016 general set and evidently, incorporating the C-C extended atom type (EAT) features led to an improvement in performance, resulting in $R_p = 0.8685$.

**Fig. 8:**
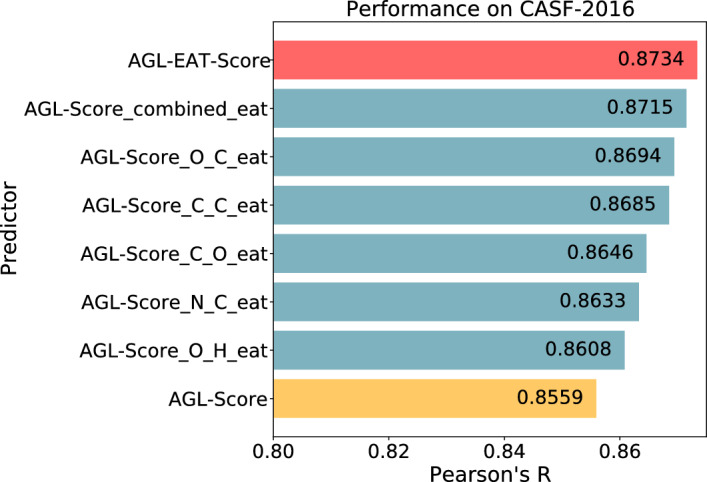
Performance comparison plot of base AGL-Score [15] (highlighted in yellow color), AGL-EAT-Score (highlighted in red color), and the modified AGL-Score with different extended atom type interaction features from AGL-EAT-Scre (highlighted in blue colors) on the PDBbind v2016 General set

We performed similar feature replacements for O-C, N-C, C-O, and O-H interactions as well, and the modified feature sets were used for test dataset predictions. A performance comparison of these experiments is presented in Fig. 8. In our final set of experiments, we replaced all the base AGL-Score C-C, O-C, N-C, and O-H interaction features with the corresponding AGL-EAT-Score extended atom type features for prediction purposes, resulting in improved performance with an $R_p = 0.8715$, named as AGL-Score-combined-eat features in Fig. 8, indicates a significant enhancement. These results demonstrate the benefits of incorporating the extra level of detail in atom-type interactions. The best performance of the model is achieved when all the extended atom types of all the element types are considered.

Finally, we compare the AGL-EAT-Score with our previously developed GGL-Score [14] to assess the robustness of algebraic graph features against geometric graph features. AGL-EAT-Score shows notable advantages in predicting binding affinities for protein-ligand complexes. In the CASF 2013 benchmark, AGL-EAT-Score achieved a Pearson R of 0.845, closely matching GGL-Score’s 0.848. Both models performed equally well in the CASF 2016 benchmark, with a Pearson R of 0.873. However, AGL-EAT-Score outperformed GGL-Score in the CatS dataset, recording a Kendall’s Tau of 0.552 and Spearman’s Rho of 0.742, compared to GGL-Score’s 0.487 and 0.674, respectively. This indicates AGL-EAT-Score’s superior ability to capture complex molecular interactions. The foundation of AGL-EAT-Score in algebraic graph theory enables a deeper analysis of structural and algebraic connectivity, revealing insights that geometric representations may overlook. This enhanced modeling capability not only improves predictive accuracy in specific contexts but also underscores AGL-EAT-Score’s adaptability in addressing the intricacies of molecular interactions. Overall, these factors position AGL-EAT-Score as a valuable tool for binding affinity prediction.

## Conclusion

In this work, we propose a novel model named Algebraic Graph Learning with Extended Atom-Type Scoring Function (AGL-EAT-Score), which exhibits high accuracy in capturing protein-ligand interaction information. This model is distinguished by its innovative integration of extended atom-type multiscale weighted colored subgraphs and algebraic graph learning, enabling a detailed and sophisticated representation of molecular interactions. The AGL-EAT-Score has demonstrated its efficacy in accurately predicting ligand-receptor binding affinities, showcasing superior performance compared to both traditional and contemporary machine learning-based scoring functions. This was evidenced through extensive evaluations using benchmark datasets such as CASF-2016, CASF-2013, and the CatS dataset.

To further validate the robustness and address concerns of overfitting in machine learning-based scoring functions, we tested the performance of the proposed AGL-EAT-Score against redundant and non-redundant data built on the PDBbind general set v2016 and the CatS dataset. The model’s performance, consistent with the level of training data information, confirms the necessity of incorporating extended atom-type information rather than relying solely on basic element types.

As the field of drug design continues to progress, the proposed AGL-EAT-Score is positioned as a robust, innovative, and essential tool for describing the complex landscape of molecular interactions, thereby contributing significantly to advancements in pharmaceutical research.

## Supplementary Information

Supplementary material 1.

## Data Availability

The source code is available at the Github repository: https://github.com/MathIntelligence/AGL-ETA-Score-Open.
