# Supplementary material for: The algebraic extended atom-type graph-based model for precise ligand–receptor binding affinity prediction
Source: J Cheminform. 2025 Jan 22;17:10. doi: 10.1186/s13321-025-00955-z (PMC11756177; doi:10.1186/s13321-025-00955-z)
Supplement: Supplementary file 1 — Supplementary material 1. [file 13321_2025_955_MOESM1_ESM.pdf]

Supporting Information: The Algebraic Extended Atom-type Graph-Based Model for Precise Ligand–Receptor Binding Affinity Prediction

Farjana Tasnim Mukta[1], Avery Meyer[1], Masud Rana[2], Sally Ellingson[3], Duc Nguyen[1]
[1]Department of Mathematics, University of Kentucky, Lexington, 40506, KY, USA
[2]Department of Mathematics, Kennesaw Sate University, Kennesaw, 30144, GA, USA
[3]Division of Biomedical Informatics, College of Medicine, University of Kentucky, Lexington, 40506, KY, USA

October 7, 2024

1 Cross-validation (CV) Performances of AGL-EAT-Score model for different PDBbind benchmark datasets and CatS dataset

A detailed discussion of optimized hyperparameters and the model's performances on each of the datasets used in this study has been documented in Figures S1,S2, and S3. For the CASF-2016 benchmark dataset, the best models are obtained to be AGL-EAT$^{\text{Adj}}_{\Phi_E,16.5,3.0}$ and AGL-EAT$^{\text{Lap}}_{\Phi_E,19.5,2.5}$ as presented in Figure S1 a and b. The median Pearson's correlation coefficient is $R_p = 0.796$ and R_p of 0.795 for the best models reported. According to the five-fold CV performances presented in Figure S2, the best models of the CASF-2013 benchmark dataset are AGL-EAT$^{\text{Adj}}_{\Phi_E,5.5,2.0}$ and AGL-EAT$^{\text{Lap}}_{\Phi_E,4.5,2.0}$ with optimized exponential kernel parameters $\kappa = 5.5$, $\tau = 2.0$ with the Adjacency matrix and $\kappa = 4.5$, $\tau = 2.0$ with the Laplacian matrix. The median $R_p = 0.795$ and $R_p = 0.796$ for the corresponding best models with the optimal kernel parameters. Finally, Figure S3 illustrates the five-fold CV performances for CatS dataset. The best models for this dataset are AGL-EAT$^{\text{Adj}}_{\Phi_E,5.5,2.0}$ and AGL-EAT$^{\text{Lap}}_{\Phi_E,4.5,2.0}$ with median Kendall's $\tau = 0.0.57837$ and 0.57305 respectively.

Figure S1 illustrates the median Pearson correlation (R_p) of the five-fold CV of AGL-EAT-Score models on the CASF-2016 benchmark dataset.

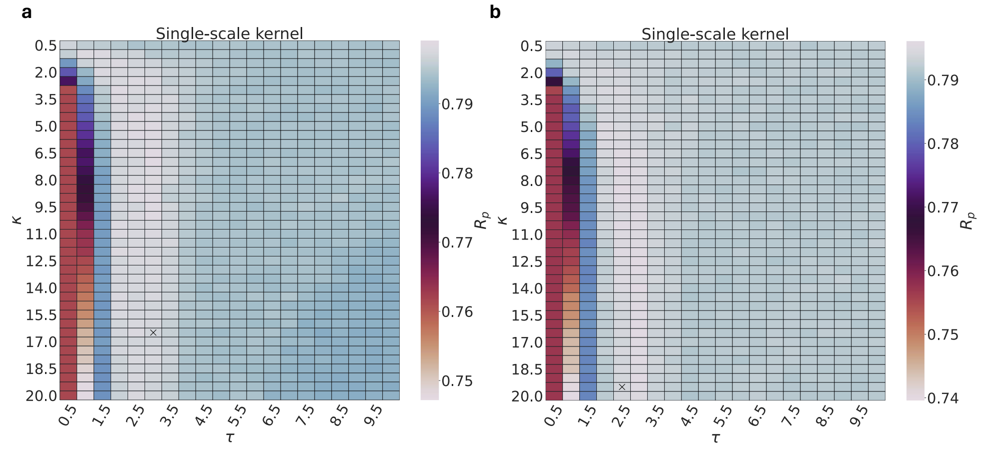

Figure S1: The optimized kernel parameters for the algebraic graph learning model with multiple atom types on the CASF-2016 dataset are indicated by 'x' marks, representing the best parameter. The optimal parameters for (a) single-scale exponential kernel model with the Adjacency matrix are $\kappa = 16.5$ and $\tau = 3.0$ with a median Pearson's correlation coefficient $R_p = 0.796$ and (b) the optimal kernel parameters for the single-scale exponential kernel model with the Laplacian matrix are $\kappa = 19.5$ and $\tau = 2.5$.

Figure S2 illustrates the median Pearson correlation (R_p) of the five-fold CV of AGL-EAT-Score models on the CASF-2013 benchmark dataset.

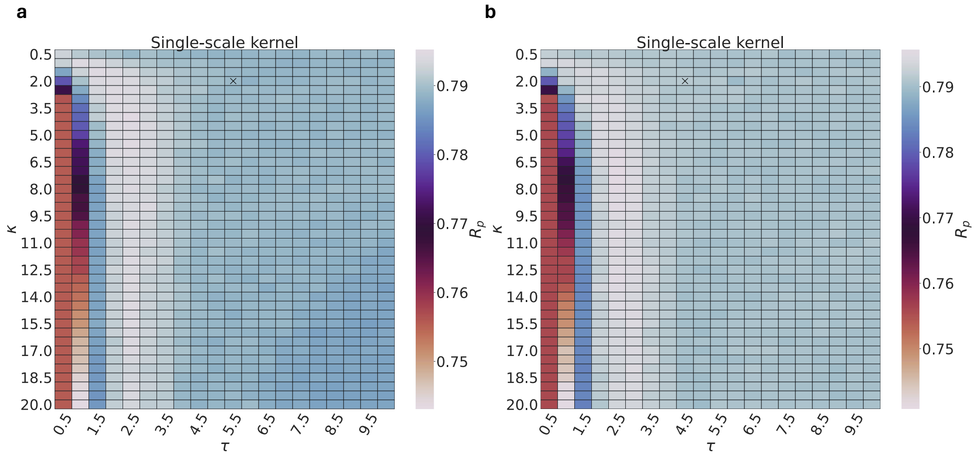

Figure S2: The optimized kernel parameters for the algebraic graph learning model with multiple atom types on the CASF-2013 dataset are indicated by 'x' marks, representing the best parameter. The optimal parameters for (a) single-scale exponential kernel model with the Adjacency matrix shows optimal kernel parameters: $\kappa = 5.5$ and $\tau = 2.0$, resulting in a median Pearson's correlation coefficient $R_p = 0.795$ and, (b) the single-scale exponential kernel model with the Laplacian matrix has optimal kernel parameters $\kappa = 4.5$ and $\tau = 2.0$, delivering a median $R_p = 0.796$.

Figure S3 illustrates the median Kendall's tau (τ) of the five-fold CV of AGL-EAT-Score models on the CatS dataset.

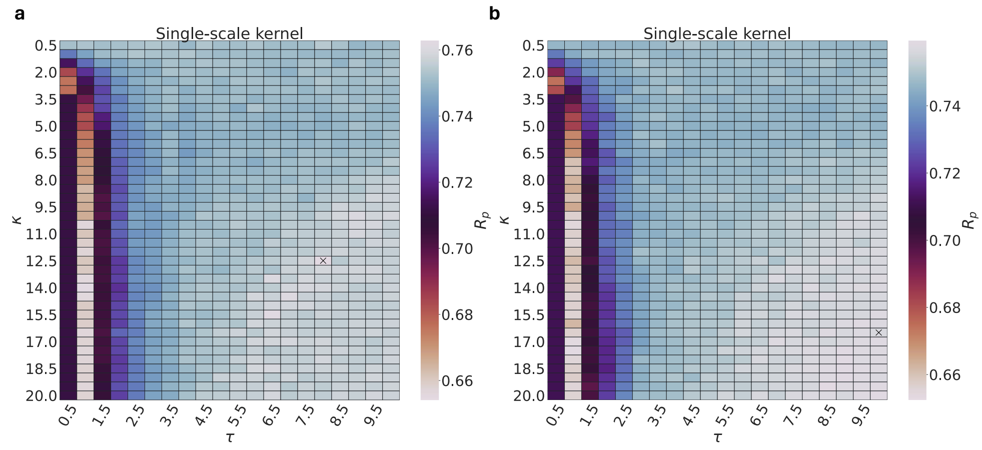

Figure S3: The optimized kernel parameters for the algebraic graph learning model with multiple atom types on the CatS dataset are indicated by 'x' marks, representing the best parameter. The optimal parameters for (a) single-scale exponential kernel model with the Adjacency matrix are $\kappa = 12.5$ and $\tau = 8.0$ with a median Kendall's $\tau = 0.57837$ and, (b) single-scale model with the Laplacian matrix are $\kappa = 16.5$ and $\tau = 10.0$ with a median Kendall's $\tau = 0.57305$.

2 Summary of Non-redundant and Redundant Training Sets for Different Similarity Cutoffs

Table S1: Training time (hr:min:s) comparison

Training set	Models		
	AGL-Score	EIC-Score	AGL-EAT-Score
PDBbindv2016 refined set	00:01:47	00:01:52	00:12:58
PDBbindv2016 general set	00:09:35	00:09:56	00:50:08

Table S2: Summary of **non-redundant complexes** of different similarity cutoffs for PDBbind v2016 GeneralSet (excluding refined set and coreset) and PDBbind v2016 RefinedSet (excluding coreset)

Similarity Cutoff	Refined Set	General Set
70% cutoff	671	6715
75% cutoff	1201	7940
80% cutoff	2040	9487
85% cutoff	2767	10920
90% cutoff	3227	11973
95% cutoff	3642	12742

Table S3: Summary of **redundant complexes** of different similarity cutoffs for CatS train vs test set

Similarity Cutoff	Redundant complexes
45%	428
50%	414
55%	356
60%	324
65%	301
70%	270
75%	226
80%	168
85%	98
90%	52
